# Vaccine-preventable diseases in migrants in Europe: a systematic review

**DOI:** 10.1016/j.vaccine.2025.127788

**Published:** 2025-10-24

**Authors:** Rae Halliday, Beatriz Morais, Oumnia Bouaddi, Anna Deal, Darlington Faijue, Sainabou Bojang, Sally Hargreaves

**Affiliations:** a The Migrant Health Research Group, Institute for Infection and Immunity, School of Health and Medical Sciences, City St George's, University of London, London, United Kingdom; b International School of Public Health, Mohammed VI University of Sciences and Health, Casablanca, Morocco; c Department of Public Health and Clinical Research, Mohammed VI Center for Research and Innovation, Rabat, Morocco

**Keywords:** Vaccination, Vaccine-preventable diseases, Outbreaks, Europe, Migrants, Asylum seekers

## Abstract

**Background:**

Migrants in Europe often face barriers to vaccination, increasing their vulnerability to vaccine-preventable diseases (VPDs). Despite policies promoting catch-up immunisation on arrival, data on VPD burden and outcomes among migrants remain limited. This systematic review synthesises evidence on the prevalence, incidence, mortality, and outcomes of VPDs in migrants across EU/EEA countries, UK, and Switzerland.

**Methods:**

We searched Medline, Embase, Global Health, and grey literature sources (including websites of national public health organisations and agencies) for primary studies and reports on VPD cases among migrants (foreign-born individuals) in 32 European countries, published between January 2010 and April 2024. Data on demographics, VPD type, vaccination status, and outcomes were extracted. We focused on diphtheria, measles, mumps, pertussis, rubella, and tetanus. Study quality was assessed using Joanna Briggs Institute tools.

**Results:**

Fifty-seven studies met inclusion criteria, reporting 1950 VPD cases in migrants across 16 countries (2010–2024). Most studies were in Germany (*n* = 12), Spain (*n* = 11), Switzerland (*n* = 8), Greece (*n* = 6), and the UK (*n* = 7). Reported cases included: measles (*n* = 992; 50.8 %), diphtheria (*n* = 546; 28.0 %), pertussis (*n* = 267; 13.7 %), and mumps (*n* = 137; 7.0 %). No cases of rubella or tetanus were reported. Migrants affected mainly included asylum seekers (*n* = 23 studies), refugees (*n* = 6), labour migrants (n = 2). Six studies from Greece, Germany, and Spain accounted for 1942 cases (99.6 %). Over half of diphtheria cases (*n* = 307; 55.4 %) occurred in reception centres. Diphtheria primarily affected adolescents and adults (*n* = 10 studies), while measles cases were mostly in children. Migrants from the Eastern Mediterranean and Africa were disproportionately affected by diphtheria. Non-EU/EEA European migrants (WHO EUR), mainly from Bosnia and Herzegovina and Serbia, accounted for most measles cases (87 %), and non-European migrants were from Somalia (*n* = 112), Afghanistan (*n* = 94), Eritrea (*n* = 76), and Syria (*n* = 64). Vaccination status was unknown or unreported in over 60 % of cases. Five VPD related deaths were reported of which 4/5 were due to measles.

**Conclusion:**

Migrants are at increased risk of VPDs due to gaps in vaccination. Strengthening catch-up vaccination, particularly in adolescents and adults, and improving data collection are essential next steps.

## Highlights

• Migrants are considered an under-vaccinated group with increased risk of vaccine-preventable diseases (VPDs).
• We found that most VPD cases in migrants in Europe were measles and diphtheria, mainly in Germany, Spain, and Greece.
• Vaccination status was unknown in over the majority of reported VPD cases.
• There is an urgent need to strengthen catch-up vaccination efforts particularly among adult and adolescent migrants.

## 1 Introduction

European countries have seen an increase in international migration over the past decades and have become host countries to diverse migrant groups, including labour migrants, asylum seekers, and refugees, in addition to internal migrants [1]. In 2023, 9 % of all EU inhabitants (42.4 million people) were born outside the EU [2]. In 2022, the continent hosted 12.4 million refugees and 1.3 million asylum seekers [3], often coming from countries with constrained health systems stretched by decades of armed conflict and economic crisis [4]. Worldwide, migrants are considered an under-immunised group for key vaccine-preventable diseases (VPDs) due to missed doses in their home countries as children, limited access to vaccination services in destination countries, and misalignment with host country schedules [5]. A global systematic review comparing vaccination coverage in adult and child migrants to host populations found that migrants were half as likely to be vaccinated than host populations [6].

The World Health Organization (WHO) Immunization Agenda 2030 and the European Vaccine Action Plan both emphasise the importance of universal access to vaccination across the life course for all groups equally, including migrants [7,8]. This also includes adolescent and adult migrants who will likely have missed vaccines as children in their home countries, due to weak vaccination systems, and then have missed vaccines, doses, and boosters during the migration process. Recently, guidelines published by the European Centre for Disease Prevention and Control (ECDC) have recommended catch-up vaccination for adult, adolescent, and child migrants arriving in European countries and called for healthcare professionals to consider administering key vaccines (measles, mumps, rubella, diphtheria, tetanus, and polio containing vaccines) to adult migrants with uncertain vaccination status or no recorded history of vaccination [9]. However, multiple studies reveal that migrants, refugees, and asylum seekers in Europe, both children and adults, still experience suboptimal access to routine and catch-up vaccination services due to persistent barriers. For instance, a study involving 16,701 migrant children in Denmark reported lower vaccine coverage for all routine childhood vaccines in the national immunisation schedule compared to Danish-born children, particularly for diphtheria, tetanus, and pertussis (DTP) vaccines [6]. Similarly, pooled immunity levels for diphtheria, measles, and mumps were found to be far below herd immunity thresholds (HIT) in a systematic review of 75,089 adult, child, and adolescent migrants across 14 countries globally, highlighting that they are an under-immunised group who could benefit from catch-up vaccination on arrival [10].

In the European context, extensive research has identified key barriers to vaccine uptake among migrant groups that limit the translation of policy intentions into actual utilization and uptake of vaccination services. These include negative perceptions of health systems, poor information about vaccination pathways, a sense of marginalisation and disengagement, as well as persistent practical and logistical challenges [11,12]. Poor vaccination uptake, low immunity, and the often-unsanitary living and working conditions (particularly for asylum seekers, refugees and undocumented migrants) suggest that some migrants in Europe remain at risk of VPD outbreaks. In fact, migrants have been affected by multiple outbreaks in European countries. A regional review found that between 2000 and 2019, there were 47 distinct VPD outbreaks across 13 European countries, involving over 9400 migrants [13]. These outbreaks mainly included measles, hepatitis A, rubella, and mumps, with most occurring in temporary refugee camps or shelters.

Despite evidence of migrants' vulnerability to VPDs, data on the burden of VPDs among migrants remains scarce, as information on country of origin, migrant status, and vaccination status is not systematically collected. For instance, previous reviews do not capture which migrant demographic groups including age categories and countries of origin are more affected by VPDs, nor the vaccination status of migrants involved in VPD outbreaks [13], information on which is needed to develop more tailored vaccination initiatives targeting specific under-immunised groups. Additionally, other morbidity and mortality outcomes, such as long-term health consequences, have not been thoroughly investigated. Thus, we conducted a systematic review to synthesise evidence on the burden of VPDs among migrants in EU/EEA countries, Switzerland, and the UK including prevalence, incidence, mortality, and longer-term outcomes across migrant groups and socio-demographic characteristics.

## 2 Methods

We conducted a systematic review to synthesise evidence on cases of VPDs in migrants in Europe between 2010 and 2024. This review is reported according to the Preferred Reporting Items for Systematic Reviews and Meta-Analyses (PRISMA) Statement 2020 [14] and has been registered on PROSPERO (CRD42024397113).

### 2.1 Search strategy

We searched three electronic databases; Medline, Embase and Global Health for primary research articles published between 1st January 2010 and 30th April 2024 in any language, reporting data on VPD cases in migrants in the EU/EEA, Switzerland and the UK. Additionally, we hand-searched websites for relevant data, including the Robert Koch Institute website (Germany) and websites of national public health organisations and agencies in the EU/EEA, Switzerland and the UK. The search strategy combined free text and subject heading terms for migrant, VPDs, and Europe (see Table S1 – Search strategy in supplementary data).

### 2.2 Study inclusion and exclusion criteria

We included primary research studies of any design published between 1st January 2010 and April 2024 in any language, reporting on cases of VPDs in migrants (defined as any individual born outside of the country in which data were collected) of all ages in the EU/EEA and Switzerland and the UK (Austria; Greece; Norway; Belgium; Hungary; Poland; Bulgaria; Iceland; Portugal; Cyprus; Ireland; Romania; Croatia; Italy; Slovakia; Czech Republic; Latvia; Slovenia; Denmark; Liechtenstein; Spain; Estonia; Lithuania; Sweden; Finland; Luxembourg; France; Malta; Germany; and the Netherlands). We included papers reporting data on the following VPDs: diphtheria, measles, mumps, pertussis, rubella, and tetanus. The primary outcome was cases of VPDs in migrants. Where available, data on mortality, long-term sequelae, migrant subpopulations, vaccination status, or immunity status were collected. National registry data and VPD incidence within host populations were collected to contextualise migrant data within the discussion.

Studies were excluded if they did not report data disaggregated by migrant status or country of origin or did not meet our country and VPD definitions. All identified records were imported into the application Rayyan [15], and duplicates were removed. Two independent reviewers screened titles, abstracts, and full texts for eligibility. The reviewers also independently reference-checked all included studies and relevant systematic reviews to identify eligible studies.

### 2.3 Data extraction

Four reviewers performed data extraction using an Excel sheet with pre-defined data points. The following information was extracted: author, publication date, VPD, country of study, region of study, study setting (primary, secondary or tertiary healthcare, community, specialist, migrant centre), number of cases, population size, prevalence, mortality, long-term sequelae, migrant subpopulation (country of origin, migrant status [e.g., refugee, asylum seeker, undocumented, economic migrant, etc.], age group [child 0–9, adolescent 10–18, adult 18+], sex, time in the host country), diagnostic method, index case, vaccination history, and genotype (pathogen genetic variant/strain, where available).

### 2.4 Quality assessment

Critical appraisal of included studies was carried out independently by two reviewers using the Joanna Briggs Institute (JBI) critical appraisal tools [16]. Studies were assessed using the JBI ‘Checklist for Case Series’ or ‘Checklist for Case Reports.’ For each criterion, studies were assigned scores of 0–2 points: 2 points for high quality, 1 point for medium-low quality, and 0 points for absence of reporting. A total score of 16 or 20 was attainable, with studies scoring over 70 % considered at low risk of bias, 50–70 % considered at moderate risk of bias, and studies scoring less than 50 % considered at high risk of bias.

## 3 Results

### 3.1 Overview of included studies

**Fig. 1:**
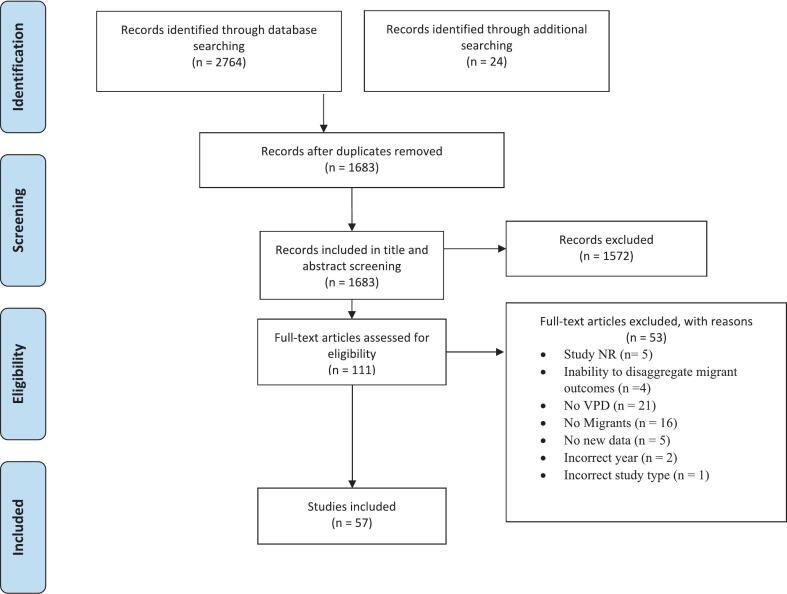
PRISMA flowchart.

**Fig. 2:**
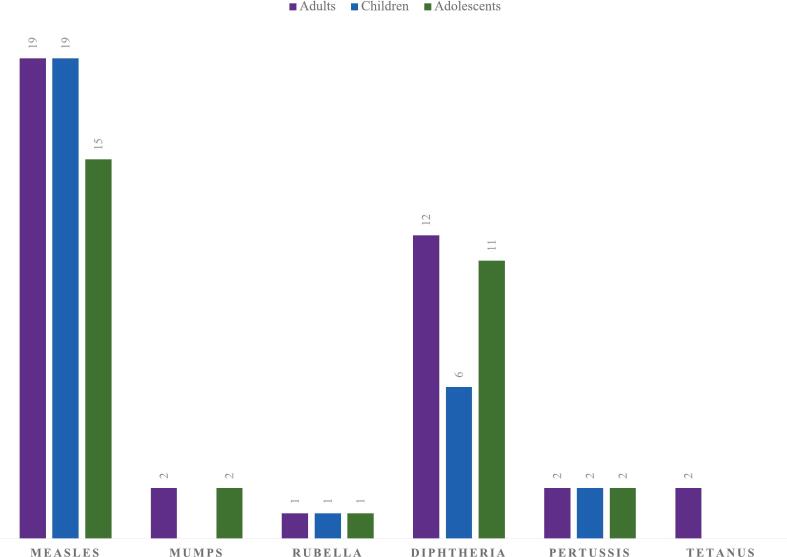
Number of studies reporting VPD cases among migrants across different age groups.

We identified 2764 database records and 24 grey literature records and screened them for eligibility. Of the 111 full-texts screened, 57 studies were included in the final analysis, involving 1950 adult and child migrants across 16 countries (Fig. 1 – PRISMA Flowchart). Most studies were in Germany (*n* = 12); Spain (*n* = 11); Switzerland (*n* = 8); Greece (*n* = 6) and the UK (*n* = 7). Thirteen studies were epidemiological reports, nine were case reports, 2 studies were cross-sectional, 2 studies were retrospective cohort studies. The most frequently reported VPDs included measles (*n* = 28 studies); diphtheria (*n* = 11); pertussis (*n* = 9); mumps (n = 9), rubella (*n* = 2); tetanus (n = 2). Regarding migrant status, studies mainly reported on asylum seekers (*n* = 23), refugees (*n* = 6), labour migrants (n = 2), students (n = 1), and tourists (n = 2), while 25 studies did not specify the type of migrants. Detailed characteristics of included studies can be found in Table 1. Socio-demographic characteristics of included VPD cases can be found in Table 2. Age and country of origin were not reported for all migrants in all studies. However, overall studies reported on migrants from all age groups and all WHO global regions. Fig. 2 demonstrates age groups reported on across all included studies.

**Table 1:** Characteristics of and quality of included studies.^a^

Author, Year	Country	Disease(s)	Study design	Study setting	Migrant type	Outbreak	Outcomes	Medium
Alberer A. 2018 [1]	Germany	Measles, Pertussis	Cross-sectional	Specialist, Migrant Centre	Refugees and asylum seekers	Yes	N of cases	Low
Badenschier 2022 [2]	Germany	Diphtheria	Outbreak report	Migrant centre	NR	Yes	N of cases; Case characteristics	Low
Barcelona Public Health Agency 2010 [3]	Spain	Pertussis, Mumps, Measles	Epi. report	NR	NR	Yes	N of cases	Medium
Barcelona Public Health Agency 2011 [4]	Spain	Pertussis, Mumps, Measles	Epi. report	NR	NR	Yes	N of cases	High
Barcelona Public Health Agency 2012 [5]	Spain	Pertussis, Mumps, Measles	Epi. report	NR	NR	Yes	N of cases	Medium
Barcelona Public Health Agency 2013 [6]	Spain	Pertussis, Mumps, Measles	Epi. report	NR	NR	Yes	N of cases	Medium
Barcelona Public Health Agency 2014 [7]	Spain	Pertussis, Mumps, Measles	Epi. report	NR	NR	Yes	N of cases	Low
Barcelona Public Health Agency 2015 [8]	Spain	Pertussis, Mumps	Epi. report	NR	NR	Yes	N of cases	High
Barcelona Public Health Agency 2016 [9]	Spain	Pertussis, Mumps	Epi. report	NR	NR	Yes	N of cases	Low
Barrett 2018 [10]	Ireland	Measles	Outbreak report	Secondary	NR	Yes	N of cases	Medium
Bloch-Infanger 2017 [11]	Switzerland	Diphtheria	Case report	Tertiary	Refugees and asylum seekers	No	N of cases	Medium
Brugueras 2019 [12]	Spain	Pertussis	Cross-sectional	Community	NR	No	N of cases	Medium
Chaud 2017 [13]	France	Measles	Outbreak report	Specialist	Refugees and asylum seekers	Yes	N of cases	High
ECDC 2024 [14]	Europe	Diphtheria	Epi. Report	Migrant Centers	Migrants	Yes	N of cases; Deaths	Medium
Filia 2016 [15]	Italy	Measles	Outbreak report	Secondary	NR	Yes	N of cases	Low
Fredlund 2011 [16]	Sweden	Diphtheria	Case Report	Primary, Secondary	Refugees and asylum seekers	No	N of cases	Medium
Galfo 2023 [17]	Italy	Diphtheria	Case report	Secondary	NR	No	Case characteristics	Medium
Georgakopoulou 2018 [18]	Greece	Measles	Outbreak report	Community, Migrant Centre	Refugees and asylum seekers	Yes	Incidence; Deaths; Vaccine history and coverage	Medium
Gianniki 2021 [19]	Greece	Measles	Epi. Report	Primary	Refugees	Yes	N of cases; Case characteristics; Deaths	Medium
Gold 2010 [20]	Germany	Measles	Outbreak report	Primary, Secondary	Labour	Yes	N of cases	Medium
Grammens 2017 [21]	Belgium	Measles	Outbreak report	Secondary	NR	Yes	N of cases	High
Huhulescu 2014 [22]	Austria	Diphtheria	Case Report	Secondary	NR	No	Case characteristics	Low
Jacquinet 2023 [23]	Belgium	Diphtheria	Epi. Report	Migrant Centre, Community	NR	Yes	N of cases; Case characteristics; Vaccine history	High
Jaton 2016 [24]	Denmark, Germany, Sweden, Switzerland	Diphtheria	Outbreak report	NR	Refugees and asylum seekers	Yes	N of cases; Case characteristics	High
Jones 2016 [25]	France	Measles	Outbreak report	Specialist, Secondary	Refugees and asylum seekers	Yes	N of cases; Case characteristics Vaccine history	Medium
Kofler 2022 [26]	Switzerland	Diphtheria	Outbreak report	Migrant centre	Asylum seekers	Yes	N of cases; Case characteristics	Low
Kolios 2017 [27]	Switzerland	Diphtheria	Case report	Migrant Centre	Refugees and asylum seekers	No	N of cases; Case characteristics; Vaccine history	Low
Kuhne 2016 [28]	Germany	Measles, Mumps	Outbreak report	Secondary	Refugees and asylum seekers	Yes	N of cases	Medium
Lanini 2014 [29]	Italy	Measles	Outbreak report	Community, Secondary	Tourist/labour	Yes	N of cases	High
Mangion 2023 [30]	Belgium	Diphtheria	Epi report	Migrant Centre	NR	No	Incidence; Case characteristics	Medium
Mankertz 2011 [31]	Germany	Measles	Outbreak report	Migrant Centre	NR	Yes	N of cases, Case characteristics	Medium
Marchant 2016 [32]	UK	Rubella	Case report	Secondary, Primary	NR	No	Long-term sequelae	Low
Meinel 2016 [33]	Germany, Switzerland	Diphtheria	Outbreak report	Migrant Centre	Refugees and asylum seekers	Yes	Vaccine history	High
Melidou 2012 [34]	Greece	Measles	Outbreak report	Secondary, Migrant Centre	NR	Yes	Incidence; Deaths	Low
Nic Lochlainn 2016 [35]	The Netherlands	Measles	Outbreak report	NR	NR	Yes	N of cases; Case characteristics	Medium
Nordbo 2016 [36]	Norway	Mumps	Outbreak report	Secondary	Student	Yes	Vaccine history	Medium
O'Boyle 2023 [37]	UK	Diphtheria	Epi. Report	NR	NR	Yes	N of cases; Case characteristics; Vaccine history	Medium
Pervanidou 2010 [38]	Greece	Measles	Outbreak report	Primary, Secondary	Labour	Yes	Incidence; Deaths; Vaccine history	High
PHE 2018 [39]	UK	Tetanus	Epi. report	Secondary	NR	No	N of cases; Vaccine history	Medium
PHE 2019 [40]	UK	Diphtheria	Epi. report	Secondary	NR	No	N of cases; Vaccine history	Low
PHE 2020 [41]	UK	Tetanus	Epi. report	Secondary	NR	No	N of cases; Vaccine history	Medium
Plachouri 2018 [42]	Greece	Measles	Outbreak report	Primary, Secondary	NR	Yes	Vaccine history	Medium
Pohl 2017 [43]	Switzerland	Diphtheria	Retrospective	Tertiary	Refugees and asylum seekers	No	Vaccine history	Medium
Rigo 2012 [44]	Hungary	Measles	Case report	Secondary	NR	Yes	N of cases	Medium
Roggendorf 2012 [45]	Germany	Measles	Outbreak report	NR	NR	Yes	Vaccine history	Medium
Salamoni 2018 [46]	Switzerland	Measles	Case report	Secondary	NR	No	Deaths; Long-term sequelae; Vaccine history	High
Sane 2016 [47]	Finland	Diphtheria	Case report	Migrant Centre, Secondary	Refugees and asylum seekers	No	Vaccine history	Low
Scheifer 2019 [48]	France	Diphtheria	Case report	Migrant Centre, Secondary	Refugees and asylum seekers	No	Vaccine history	High
Seppala 2017 [49]	Finland	Measles	Outbreak report	Secondary	Tourist	Yes	Vaccine history	High
Seppala 2019 [50]	Spain	Rubella	Retrospective	Secondary	NR	No	Incidence; Vaccine history	Medium
Sing A. 2023 [51]	Germany	Diphtheria	Outbreak report	Reception Centre	NR	Yes	N of cases; Case characteristics	Low
Spielberger 2022 [52]	Germany	Diphtheria	Retrospective	Migrant Centre	NR	Yes	N of cases; Case characteristics	Low
Takla 2012 [53]	German	Measles	Comparative	Migrant Centre	Refugees and asylum seekers	Yes	N of cases	Medium
Torner 2013 [54]	Spain	Measles	Outbreak report	NR	NR	Yes	N of cases	High
Traugott 2022 [55]	Austria	Diphtheria	Case report	Migrant Centre	NR	No	N of cases; Deaths	Medium
Vainio 2011 [56]	Norway	Measles	Outbreak report	NR	NR	Yes	Vaccine history	Medium
Vaquero 2018 [57]	Spain	Diphtheria	Case report	Secondary	NR	No	Case characteristics	Low
Werber 2017 [58]	Germany	Measles	Outbreak report	Migrant Centre	Refugees and asylum seekers	Yes	Incidence; Deaths; Vaccine history	High

a NR = Not reported.

**Table 2:** Socio-demographic characteristics of VPD cases in migrants in Europe.^a^

Author, Year Country	Setting	Migrant (N)	Non-migrant (N)	Age groups	WHO region^b^ of origin of migrants					
					EUR	EMR	AFR	AMR	WPR	SEAR
**Measles**										
Alberer A. 2018 [1] Germany	Specialist, Migrant Centre	5		Children; Adults						
Chaud 2017 [13] France	Specialist	9	4	Adult		**X**	**X**			
Georgakopoulou 2018 [18] Greece	Community, Migrant Centre	316	1910 (roma), 924 (non-minority nationals)	All	**X**					
Jones 2016 [25] France	Specialist, Secondary	16		All						
Kuhne 2016 [28] Germany	Secondary	82								
Melidou 2012 [34] Greece	Secondary, Migrant Centre	126								
Pervanidou 2010 [38] Greece	Primary, Secondary	39	87	All	**X**					
Plachouri 2018 [42] Greece	Primary, Secondary	4	137 (106 roma, 31 non-minority Greek)	All						
Rigo 2012 [44] Hungary	Secondary	5		Children; Adults	**X**					
Roggendorf 2012 [45] Germany	NR	2	6	All	**X**					
Salamoni 2018 [46] Switzerland	Secondary	1		Children		**X**				
Seppala 2017 [49] Finland	Secondary	4	2	Adults	**X**					
Takla 2012 [53] German	Migrant Centre	8	0	All	**X**	**X**	**X**		**X**	**X**
Vainio 2011 [56] Norway	NR	8	2	All	**X**		**X**			
Werber 2017 [58] Germany	Migrant Centre	146	1101	All	**X**	**X**				
Barrett 2018 [10] Ireland	Secondary	11	25	All						
Barcelona Public Health Agency 2011–2016 [[3], [4], [5], [6], [7], [8], [9]] Spain	NR	45	88	NR	**X**		**X**	**X**	**X**	**X**
Filia 2016 [15] Italy	NR	3	64 (40 Roma/Sinti)	All			**X**			**X**
Grammens 2017 [21] Belgium	NR	NR	288 (177 data provided)	All	**X**					
Gold 2010 [20] Germany	Primary, Secondary	10	38	All	**X**					
Lanini 2014 [29] Italy	Community, Secondary	13	14	All	**X**			**X**	**X**	**X**
Mankertz 2011 [31] Germany	NR	42	20	Children	**X**	**X**				
Nic Lochlainn 2016 [35] The Netherlands	NR	1	32	All					**X**	
Torner 2013 [54] Spain	NR	78	227	All						
Gianniki S. 2021 [19] Greece	Primary	18	64 Greek, 429 Roma	NR						

**Mumps**										
Kuhne 2016 [28] Germany	Secondary	2		NR						
Nordbo 2016 [36] Norway	Secondary	1	148	Adolescents; Adult						
Barcelona Public Health Agency 2010–2016 [[3], [4], [5], [6], [7], [8], [9]] Spain	Not reported	134	280	NR	**X**	**X**	**X**	**X**	**X**	**X**

**Rubella**										
Seppala 2019 [50] Spain	Secondary	5		Adolescents; Adults	**X**	**X**	**X**	**X**		
Marchant 2016 [32] UK	Secondary, Primary	1		Newborn			**X**			

**Diphtheria**										
Bloch-Infanger 2017 [11] Switzerland	Tertiary	44		Child, Adolescent, Adult			**X**			
Fredlund 2011 [16] Sweden	Primary, Secondary	2		Adult, Adolescent			**X**			
Huhulescu 2014 [22] Austria	Secondary	1		Adolescent			**X**			
Jaton 2016 [24] Denmark, Germany, Sweden, Switzerland	NR	16					**X**			
Kolios 2017 [27] Switzerland	Secondary	1	0	Adult			**X**			
Meinel 2016 [33] Germany, Switzerland	Secondary, Migrant Centre	20	5	Adolescent, Adult		**X**	**X**			
Pohl 2017 [43] Switzerland	Tertiary	2		Child, Adolescent	**X**	**X**	**X**			
Sane 2016 [47] Finland	Migrant Centre, Secondary	1		Adolescent		**X**				
Scheifer 2019 [48] France	Migrant Centre, Secondary	1	1 (48 total 2010 to 2013, 24 in 2014, 36 in 2015 in Europe)	Adult		**X**				
PHE 2019 [40] UK	Secondary	1	7	NR			**X**		**X**	
Badenschier 2022 [2] Germany	Migrant Centre	44		Child, Adolescent, Adult		**X**				
Vaquero 2018 [57] Spain	Secondary	1		Adult			**X**			
Kofler 2022 [26] Switzerland	Migrant Centers	17		Child, Adolescent, Adult		**X**				
Traugott 2023 [55] Austria	Migrant Centre	1		Adult		**X**				
Jacquinet S. 2023 [23] Belgium	Migrant Centre, Community	28 (25 cutaneous, 3 respiratory)		Child, Adolescent, Adult		**X**				
O'Boyle 2023 [37] UK	Migrant Centre	72		Child & Adolescent (39 (53 %) younger than 18), Adult		**X**				
Sing 2023 [51] Germany	Migrant Centre	167		NR		**X**				
Galfo [17] Italy	Secondary	1		35						
Spielberger 2022 [52] Germany	Migrant Centre	27		Adult		**X**				
Mangion 2023 [30] Belgium	Migrant Centre	8		Adult		**X**				
ECDC 2024 [14] Europe	Migrant Centre	92		NR						

**Pertussis**										
Alberer A. 2018 [1] Germany	Specialist, Migrant Centre	1		Child, Adolescent, Adult		**X**	**X**			
Brugueras 2019 [12] Spain	Community	122 (foreign-born), 54 unknown	1217 (native), 54 (unknown)	Child, Adolescent, Adult						
Barcelona Public Health Agency 2010–2016 [[3], [4], [5], [6], [7], [8], [9]] Spain	Not reported	144	804, 34 unknown	NR	**X**	**X**	**X**	**X**	**X**	**X**

**Tetanus**										
PHE 2020 [41] UK	Secondary	1	3	Adult						
PHE 2018 [39] UK	Secondary	1	3	Adult						

a NR = Not reported.

b WHO EUR = European Region; WHO AMR = Region of the Americas; WHO EMR = Eastern Mediterranean Region; WHO AFR = African Region; WHO WPR = Western Pacific Region; WHO SEAR = South-East Asia Region.

Overall, the quality of studies was found to be medium-low. Seventeen studies were considered at high-risk of bias, 20 were considered at medium risk of bias and only 16 were considered at low risk of bias.

### 3.2 Overall cases of VPDs

Across the included studies, 1950 unique cases of VPDs were reported. The majority of cases were reported in six studies across 3 countries (Greece, Germany and Spain).In total, there were 992 cases of measles, 546 of diphtheria, 267 of pertussis, 137 of mumps, 6 of rubella, and 2 of tetanus. Studies predominantly reported cases from secondary healthcare facilities or migrant reception centres, with many studies reporting from multiple settings without presenting disaggregated data by setting (Table 2).

#### 3.2.1 Measles

Twenty-nine studies reported 992 cases of measles in migrants versus 5468 in non-migrants across 12 EU countries between 2010 and 2020, with 20 studies related to outbreaks, predominantly in Germany [[17], [18], [19], [20], [21], [22], [23]] and Greece [[24], [25], [26], [27]], accounting for the majority of cases (*n* = 842; 84.8 %). Most cases were among refugees, asylum seekers, or individuals with unspecified status, and one case involved a tourist. Across the 21 studies that reported measles cases in both migrants and non-migrants, the proportion of migrant cases ranged from 2.8 % to 83.3 %, while the proportion of non-migrant cases ranged from 16.7 % to 97.2 %. Further, the proportion of measles cases was higher in migrants than non-migrants in 5 studies, higher in non-migrants in 13 studies, and equal in 2 studies. Regarding age groups, cases were reported among children (*n* = 19), adolescents (*n* = 16), and adults (n = 19). However, exact continuous age data in measles studies were inconsistently reported, limiting comparisons between studies. Data on the countries of origin of migrants were also inconsistently reported, with seven studies omitting such data and several others not disaggregating it by disease status. Across studies reporting case numbers by country of origin, non-EU/EEA European migrants from WHO EUR (mainly from Bosnia and Herzegovina and Serbia) accounted for the majority of cases (87 %), with cases reported in migrants residing mainly in Germany and Greece [[17], [18], [19], [20], [21],[24], [25], [26],^28^]. Measles outbreaks involving these European migrants were generally larger, with the largest event in Greece in 2017/18 involving 316 migrant cases. In contrast, despite lower case numbers, more studies reported cases among non-European migrants, mainly among refugees and asylum seekers. Among non EU/EEA European migrants, cases most frequently originated from Bosnia and Herzegovina and Serbia [21]. Non-European migrant cases were primarily reported among individuals from Somalia, Afghanistan, Eritrea, and Syria.

Vaccination history was provided in half of the studies [19,21,22,24,26,27,[29], [30], [31], [32]]. However, the majority of studies did not disaggregate vaccination status data by migrant or disease status. Across the studies that did report vaccination status among measles cases, at least 372 cases had received one or more doses of vaccine, 3094 cases were unvaccinated, and at least 75 had unknown vaccination status. This information, however, was not disaggregated by migrant status.

Measles-related deaths were reported in three studies. Four deaths occurred during a Greek outbreak in 2017/18, none involving migrants [24]. One death involved a 12-year-old Somalian-born child in Switzerland in 2018 due to subacute sclerosing panencephalitis following prior measles infection [32]. Another death was linked to a 2014/15 outbreak in a Berlin migrant center involving 146 migrant cases, but the migrant status, age, and sex of the deceased were unspecified [21].

#### 3.2.2 Mumps

Nine studies reported 137 cases of mumps in migrants (including one student) and 428 in non-migrants. Across the 7 studies that reported mumps cases in both migrants and non-migrants, the proportion of migrant cases ranged from 12.5 % to 25 %, and the proportion of non-migrant cases from 75 % to 87.5 %. In all 7 studies, the proportion of cases was higher among non-migrants than migrants. Most cases in migrants (*n* = 134; 97.8 %) were reported in Spain, [[33], [34], [35], [36], [37], [38], [39]] among migrants from WHO EUR (Germany, UK, Sweden, France, Italy, Finland, Greece, Romania and Norway), [18,33,[35], [36], [37], [38], [39], [40]], AMR (Argentina, Brazil, Cuba, USA, Ecuador, Dominican Republic, Paraguay, Bolivia, Columbia, Peru), [33,[35], [36], [37], [38]] EMR (Pakistan and Morocco), [[33], [34], [35], [36], [37], [38],^41^] AFR (Cameroon, Ethiopia, Mali), [[34], [35], [36]] SEAR (Bangladesh and India), [[33], [34], [35]] WPR (Philippines and China) [33,35,36]. Age sex, and vaccination status data were not available for any mumps cases.

#### 3.2.3 Rubella

Two studies reported on 6 cases of rubella in migrants in the UK and Spain, with no data on non-migrants [41,42]. Two of the six migrant cases were imported from Romania and Pakistan. Secondary cases occurred in migrants originating from Romania, Morocco and the Dominican Republic [41]. One of the cases was reported in a tourist. No information was found on vaccination status.

#### 3.2.4 Diphtheria

**Fig. 3:**
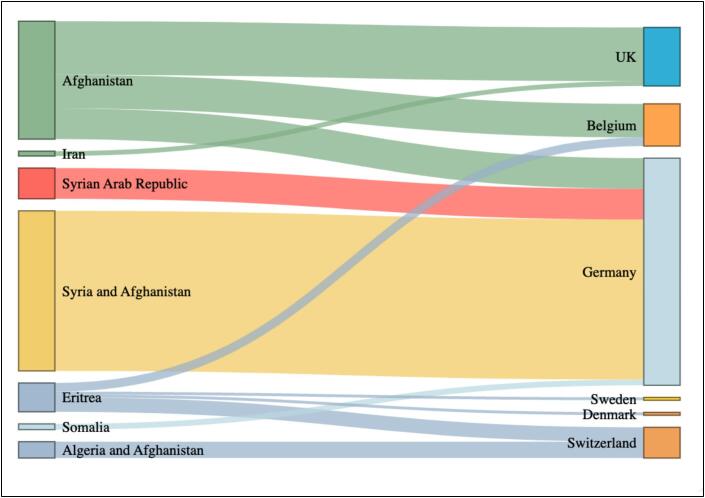
Sankey diagram of countries of origin and host countries of diphtheria cases in migrants.

Twenty-one studies reported on 547 cases of diphtheria in migrants across 11 countries (Switzerland, Sweden, Austria, Denmark, Germany, Finland, France, UK, Spain, Belgium, Italy) vs 13 non-migrants in 3 studies across 2 countries (UK and France) [[43], [44], [45]]. Most diphtheria cases in migrants (*n* = 395; 72.2 %) were reported in only three countries (Germany, Switzerland and the UK). Over half of the cases (*n* = 307; 55.4 %) were reported in migrant or refugee reception centres. In contrast with measles, diphtheria cases were predominantly reported among adolescents and adults (*n* = 10), with only four studies presenting cases of diphtheria in children [44,[46], [47], [48]]. Diphtheria cases were overwhelmingly reported among migrants originating from the WHO Eastern Mediterranean region (*n* = 388) followed by African region (*n* = 11), with Afghanistan (n = 11), Syria (*n* = 7), Eritrea (*n* = 4) and Somalia (n = 1) commonly reported as countries of origin, see Fig. 3. Only one study – a case report – reported a death related to diphtheria [49], and no study recorded vaccination status.

#### 3.2.5 Pertussis

Nine studies reported 267 cases of pertussis in migrants and 2021 in non-migrants across Germany and Spain between 2010 and 2019. Most migrant cases (99.0 %) were reported in Spain. Across the 6 studies that reported pertussis cases in both migrants and non-migrants, all showed a higher proportion in non-migrants. The proportion of migrant cases ranged from 4.3 % to 21.9 % versus 78.1 % to 95.7 % in non-migrants. Age data for all studies were poorly reported, one report provided age group data for all cases involved without disaggregation by disease status [50]. Six reports in Spain reported data on country of origin of migrants showing that all WHO regions (EUR, AMR, EMR, AFR, WPR, SEAR) were represented with the most frequently reported countries being Ecuador, Morocco and Peru. The WHO AMR was overrepresented, with 63 migrants originating from 13 countries; followed by EUR, with 35 migrants originating from 14 countries. Of migrant cases originating in the WHO AMR, most cases originated from Ecuador [51], Peru [52] or Bolivia [53]. Of European migrant cases, Romanian, French and Italian migrants represented the most common country of origin with 5, 5 and 4 migrants originating from these countries respectively. None of the studies reported vaccination status.

#### 3.2.6 Tetanus

Tetanus was not reported in any included studies from database searching. However, hand-searching of national websites yielded two reports from Public Health England, reporting cases of tetanus in non-UK born individuals in 2017 [54] and 2019 [55]. Both cases were adults, exact age, time in country and sex data were not available. One case self-reported vaccination as a child [55], however vaccination records and country of origin were not available for either case.

## 4 Discussion

This systematic review examined 1950 cases of VPDs among migrants in 16 EU/EEA countries, Switzerland, and the UK (2010–2024). Migrants from the Eastern Mediterranean and Africa were disproportionately affected by diphtheria, mainly in reception centres, and predominantly among adolescents and adults. Measles cases were mostly reported among non-EU/EEA European migrants (mainly from Bosnia and Herzegovina and Serbia) residing in Greece and Germany, while non-European cases involved migrants from Somalia, Afghanistan, Eritrea, and Syria. Vaccination history was often missing or unclear, and data on age and outcomes by migrant status were inconsistently reported. Overall, migrants remain an at-risk group for VPDs, highlighting the need for strengthened catch-up vaccination programs and improved routine data collection by migrant status.

This review found that measles affected the widest age groups of migrants, including both children and adults. This suggests that migrant children, adolescents and adults may be under immunised and at risk of measles infection. Likewise, a recent systematic review and meta-analysis in the EU, EEA, Switzerland, and the UK showed that immunity levels for measles (83.7 % vs HIT 93–95 %), mumps (67.1 % vs HIT 88–93 %), and rubella (85.6 % vs HIT 83–94 %) were all below the herd immunity thresholds (HIT), with lower immunity levels in children compared to adults for measles and mumps [10]. Although nearly all EU countries include full vaccinations for migrant children in their national immunisation plans, this review, like earlier studies, found that these policies are not well implemented [56]. Inconsistent vaccination practices, weak EU-level coordination, and poor surveillance systems make catch-up vaccination campaigns less effective. Existing evidence also shows that inclusion of migrants into national immunisation policies does not equate actual uptake and utilization of these services due to persisting individual and structural barriers other than entitlement. Research on drivers of catch-up vaccination in migrants in Europe is scarce, but some small-scale studies suggest that migrants often accept catch-up immunisation when offered [57].

Furthermore, this review has found a greater number of measles cases reported among non-EU/EEA European migrants (predominantly from Bosnia Hertzogovenia and Serbia) than non-European migrants. These outbreaks often result in larger caseloads and a higher risk of spread between migrant and non-migrant communities. In contrast, the current evidence base has been found to contain a greater number of reports on the incidence of measles in refugees and asylum seekers, despite a lower number of outbreaks associated with these migrant groups, compared to migrants of European origin. Previous studies have demonstrated an 89 % measles seropositivity (presence of antibodies) among refugees [58], however overall MCV2 coverage was estimated to be below 80 % in Romania in 2015 [59], the most common country of origin for European migrants in this review. The relationship between measles outbreaks and migrants of European origin therefore represents an important and potentially under-reported area of EU measles monitoring, with key implications for measles catch-up vaccination.

Another key finding in this review is that over 70 % of diphtheria cases were reported in just three countries, with over half occurring in reception centres and mainly in adults from the eastern Mediterranean and African regions. The subpopulation of migrants involved in diphtheria cases largely reflects the demographic of newly arrived migrants between 2014 and 2017 [60]. A previous meta-analysis showed that diphtheria immunity (defined as recorded vaccination history or laboratory confirmation) among migrants in the EU/EEA, Switzerland, and the UK was significantly below the herd immunity threshold, with pooled immunity coverage at 57.4 % (95 % CI: 43.1–71.7) [61], and immunity levels lower among adults compared to children (63.9 % vs. 76.0 %) [10]. This may be due to a lack of tailored policies and programmes for catch-up vaccination for adults, alongside a potential epidemiological shift in the incidence of VPDs among adults in LMICs (countries of origin), although robust data on this are limited [62,63] In fact, a review of EU vaccination policy and practice reported that only 16 of 32 EU countries currently offer diphtheria-containing catch-up vaccination to migrants on arrival [61]. In 2018, the ECDC released guidelines for catch-up vaccination for migrant adults with no immunisation records or uncertain status [64]. These guidelines recommend administering one dose of MMR vaccine in accordance with the MMR schedule of the host country, and vaccinating in accordance with the host country's schedule with priority given to the primary series of diphtheria, tetanus, and polio vaccines [61]. The findings of this review highlight a clear limitation of current EU migrant vaccination policies and identify a subpopulation of migrants who may be at risk, particularly those in reception centres. Improved screening of newly arrived adult migrants and the provision of easily accessible, free DTP vaccination in all EU countries is essential [61].

While the implementation bottlenecks for catch-up vaccination guideline for migrants have not been studies, and the data on vaccination status in this review was quasi absent, existing evidence suggest that migrants in Europe may not be aligned with destination country schedules.

For example, a global analysis of 12,526 UK-bound refugees from 36 countries found that only 34 % and 5 % were aligned with UK vaccination schedules for measles and diphtheria, respectively, with adults significantly less likely to be immunised on schedule [65]. In the UK as well, where national catch-up vaccination guidelines for migrants exist, a study found that less than 10 % of adults and adolescents were offered vaccines such as MMR, Td/IPV, MenACWY, and HPV [66]. Similarly, a qualitative study revealed that adult migrants were rarely offered catch-up vaccinations in the UK context [67]. The main challenges reported included low awareness, lack of trust in authorities, negative past experiences of discrimination and injustice, as well as logistical and administrative barriers [68]. These findings suggest that strengthening the implementation of ECDC guidelines for catch-up vaccination must be accompanied by efforts to raise awareness and provide incentives for healthcare professionals [9]. Moreover, these efforts should include initiatives to build trust and address migrants' concerns using appropriate information channels and culturally tailored messaging.

This review has several limitations. First, there is a significant lack of reporting across the included studies. Many studies did not disaggregate data by migrant status or used inconsistent definitions for migrant populations and poor reporting by age and gender. This inconsistency made it difficult to draw conclusions about the burden of VPDs among migrants compared to non-migrants. While we applied a broad definition of migrants to capture diverse subgroups, the available information was largely limited to refugees, asylum seekers, or those categorized under unspecified migrant groups. In contrast, labour migrants, who constitute the majority of migrants in Europe, remain underrepresented, which shows a persistent mismatch between the size of migrant populations in the region and the focus of existing studies. Evidence indicates that labour migrants, particularly in the food and agricultural sectors, often live and work in conditions conducive to the spread of VPDs, similar to asylum seekers and refugees in closed settings [69]. Moreover, undocumented and irregular migrants were entirely absent from the reviewed studies, leaving their risk of VPDs unclear, although these populations are also less likely to access health and vaccination services, which may lead to severe consequences of VPDs due to delays in care. Another major limitation is a scarcity of data on the vaccination status of migrant VPD cases with many records being unknown or incomplete. These gaps call for the need for more systematic data collection to track vaccination status and coverage in migrants for key VPDs, particularly at-risk groups. Recommendations for policy, practice and future research to address these gaps are outlined in Panel 1.

**Table:** 

**Panel 1. Implication for policy, practice and research** **Policy** - Strengthen the implementation of EU-level guidelines on catch-up vaccination for adults, adolescents and children with uncertain or incomplete vaccination records - Implement existing ECDC guidelines, offer MMR and DTP catch-up vaccination to all newly arrived child, adult and adolescent migrants, free of charge and in multiple settings - Programmes could consider targeting specific nationality groups at high risk of under-immunisation, or migrants housed in closed settings ***Practice*** - Expand current vaccine policy to ensure all migrants have equitable access, free of charge, to all vaccinations offered by national schedules, including tailored programmes for at-risk migrant groups - Streamline healthcare services to better engage with and support migrant health needs, including devising strategies to better engage migrants with primary healthcare services ***Research*** - Explore the social barriers for migrants accessing vaccination to ensure improved uptake - Strengthen European data collection, surveillance, and reporting around VPDs, disaggregating by migrant status

In conclusion, this review has highlighted that migrants may be an at-risk group for VPDs in the European context. In order to gain a fuller understanding of the relationship between VPD incidence and migration in Europe, and to more effectively inform policy, further research is required on the demographics of migrants particularly at-risk of VPD, in addition to improvements in current reporting and surveillance systems.

## CRediT authorship contribution statement

**Rae Halliday:** Writing – review & editing, Writing – original draft, Visualization, Validation, Methodology, Investigation, Formal analysis, Data curation, Conceptualization. **Beatriz Morais:** Writing – review & editing, Writing – original draft, Visualization, Formal analysis, Data curation. **Oumnia Bouaddi:** Writing – review & editing, Writing – original draft, Visualization, Validation, Formal analysis, Data curation. **Anna Deal:** Writing – review & editing, Writing – original draft, Methodology, Conceptualization. **Darlington Faijue:** Writing – review & editing, Data curation. **Sainabou Bojang:** Writing – review & editing. **Sally Hargreaves:** Writing – review & editing, Writing – original draft, Validation, Supervision, Project administration, Methodology, Funding acquisition, Data curation, Conceptualization.

## Funding

This work was funded by the NIHR (NIHR300072) and the MRC (MRC/N013638/1).

## Declaration of competing interest

The authors declare that they have no known competing financial interests or personal relationships that could have appeared to influence the work reported in this paper.

## Data Availability

The data presented in this review is available online.

## Appendix A Supplementary data

Supplementary material Search Strategy. Image 1
