# Supplementary material for: Vaccine-preventable diseases in migrants in Europe: a systematic review
Source: Vaccine. 2025 Oct 24;65:None. doi: 10.1016/j.vaccine.2025.127788 (PMC13279756; doi:10.1016/j.vaccine.2025.127788)
Supplement: Supplementary material — Search Strategy. [file mmc1.docx]

**SUPPLEMENTARY DATA**

**Table 1. Search strategy (Embase)**

| Migrant | Migrant* OR immigrat* OR emigrat* OR migrati* OR immigrant* OR emigrant* OR unaccompanied child* OR unaccompanied minor OR foreign-born* OR foreign born* OR foreign-origin OR foreign origin OR foreign* OR asylum OR asylum seek* OR asylum-seek* OR refused asylum* OR refugee* OR non-citizen* OR citizenship OR nationality* OR undocumented* OR illegal* OR non-resident* OR transient* OR country ajd3 origin* OR expat* OR newcomer* OR new-comer* OR |
| --- | --- |
| Migrant MeSH | undocumented immigrant/ or immigrant/ or migrant/ or emigrant/ or refugee/ or short distance migrant/ or long distance migrant/ or migrant worker/ |
| Cases | Prevalen* OR inciden* OR epidemiol* OR case* OR outbreak* OR mortalit* OR |
| Cases MeSH | prevalence/ or incidence/ or epidemiological data/ or epidemiology/ or case report/ or case study/ or case mix/ or epidemic/ or mortality/ or mortality rate/ or mortality risk/ |
| VPD | VPD OR vaccine-preventable disease OR measles OR Mumps OR Rubella OR Diphtheria* OR Diphtheritic OR Pertussis OR whooping cough* OR tetanus |
| VPD MeSH | measles/ or mumps/ or rubella/ or pertussis/ or diphtheria/ or tetanus/ |
| EU/EEA | Austria* OR Belgium OR Belgian OR Bulgaria* OR Cyprus OR Cypriot OR Czech OR Denmark OR Danish OR Estonia* OR Finland OR Finnish OR France OR French OR German* OR Greece OR Greek OR Hungar* OR Iceland* OR Ireland OR Irish OR Italy OR Italian OR Latvia* OR Liechtenstein OR Lithuania* OR Luxembourg* OR Malta OR Maltese OR Netherlands OR Dutch OR Norway OR Norwegian OR Poland OR Polish OR Portug* OR Romania* OR Slovakia* OR Slovenia* OR Spain OR Spanish OR Sweden OR Swedish OR Switzerland OR Swiss OR Great Britain OR British OR United Kingdom OR UK OR England OR English OR Wales OR Welsh OR Scotland OR Scottish OR EU OR EEA OR Europe* OR |
| EU/EEA MeSH | EU citizen/ or European Union/ or Eastern European/ or Northern European/ or Central European/ or Southern European/ or European/ or Western European/ |
